# Association between body mass index and *Firmicutes/Bacteroidetes* ratio in an adult Ukrainian population

**DOI:** 10.1186/s12866-017-1027-1

**Published:** 2017-05-22

**Authors:** Alexander Koliada, Ganna Syzenko, Vladislav Moseiko, Liudmyla Budovska, Kostiantyn Puchkov, Vyacheslav Perederiy, Yuriy Gavalko, Andriy Dorofeyev, Maryana Romanenko, Sergiy Tkach, Lyudmila Sineok, Oleh Lushchak, Alexander Vaiserman

**Affiliations:** 1 D. F. Chebotarev Institute of Gerontology, NAMS, Kiev, Ukraine; 2 O.O. Bogomolets National Medical University, Kiev, Ukraine; 3 Vasyl Stefanyk Precarpathian National University, Ivano-Frankivsk, Ukraine

**Keywords:** Obesity, Gut microbiota, *Actinobacteria*, *Firmicutes*, *Bacteroidetes*

## Abstract

**Background:**

Metagenomic studies confirm that obesity is associated with a composition of gut microbiota. There are some controversies, however, about the composition of gut microbial communities in obese individuals in different populations. To examine the association between body mass index and microbiota composition in Ukrainian population, fecal concentrations of *Bacteroidetes*, *Firmicutes*, *Actinobacteria* and *Firmicutes/Bacteroidetes* (F/B) ratio were analyzed in 61 adult individuals.

**Results:**

The relative abundance of *Actinobacteria* was small (5–7%) and comparable in different BMI categories. The content of *Firmicutes* was gradually increased while the content of *Bacteroidetes* was decreased with increasing body mass index (BMI). The F/B ratio also raised with increasing BMI. In an unadjusted logistic regression model, F/B ratio was significantly associated with BMI (OR = 1.23, 95% CI 1,09–1,38). This association continued to be significant after adjusting for confounders such as age, sex, tobacco smoking and physical activity (OR = 1.33, 95% CI 1,11–1,60).

**Conclusions:**

The obtained data indicate that obese persons in Ukraine adult population have a significantly higher level of *Firmicutes* and lower level of *Bacteroidetes* compared to normal-weight and lean adults.

**Electronic supplementary material:**

The online version of this article (doi:10.1186/s12866-017-1027-1) contains supplementary material, which is available to authorized users.

## Background

The epidemic of obesity around the world has become an important public health issue, with serious psychological and social consequences [1, 2]. Obesity is recognized as a multifactorial disorder which is a result of the interaction of host and environmental factors, occurring when *energy intake exceeds energy expenditure* over time [1]. The gut microbiota is known to play an important role in energy homeostasis [3, 4]. Metagenomic studies confirm that gut microbiota in obese subjects is more efficient than that in lean subjects at recovering the energy from resistant dietary components [5, 6]. Previous animal studies provided *insight into the* underlying mechanisms of that phenomenon. There are: (1) *increased caloric intake* from indigestible polysaccharides, combined with effect on hepatic de novo lipogenesis via carbohydrate and sterol response-element binding proteins; (2) enhanced cellular uptake of fatty acids and storage of triglycerides in adipocytes via suppression of intestinal expression of fasting-induced adipocyte factor which is circulating inhibitor of lipoprotein lipase; (3) suppression the skeletal muscle fatty acid oxidation through a metabolic pathway involving phosphorylation of adenosine monophosphate-activated protein kinase; and (4) interaction between short chain fatty acids products of microbial fermentation of dietary polysaccharides and G-protein-coupled receptor 41 which results in increased levels of enteroendocrine cell-derived hormone PYY, thus, reducing gut motility with subsequently increased intestinal transit time and absorption rate of short-chain fatty acids [7–9]. Microbiota can also promote obesity and metabolic syndrome by inducing low-grade inflammation [7, 10]. Currently, gut microbiota is increasingly considered as a “metabolic organ” greatly affecting the organism’s metabolism [11]. According to this point of view, there is plausible reason to suppose that differences in gut microbiota may be linked to energy homeostasis, thus predicting that obese and lean individuals have distinct microbiota composition, with measurable difference in the ability to extract energy from the food and to store those energy as the fat [12].

Presently, changes in intestinal microbial composition are believed to be an important causal factor in development of obesity [13]. The most common organisms in human gut microbiota are members of the gram-positive *Firmicute*s and the gram-negative *Bacteroidetes* phyla, with several others phyla, including the *Actinobacteria, Fusobacteria* and *Verrucomicrobia*, that are present at subdominant levels [14]. Data obtained from animal models revealed consistent differences in the two major bacterial phyla with significant increase of the *Firmicutes* and decrease of the *Bacteroidetes* levels in ob/ob compared to wild-type mice despite a similarity in their diet and activity levels [15]. Consistently with animal data, numerous human studies have consistently demonstrated that the *Firmicutes/Bacteroidetes* (F/B) proportion is increased in obese people compared to lean people, and tend to decrease with weight loss (for reviews, see [16–18]). Several studies, however, have produced conflicting results. Some investigations have failed to find significant differences in the F/B ratio between lean and obese humans at both baseline level and after the weight loss [19–24]. In some studies, the fecal concentrations of *Bacteroides* were positively correlated with body mass index (BMI) [25], and predominance of *Bacteroidetes* in overweight and obese individuals was demonstrated [26]. Most likely, these differences can be due to different environmental influences, including diet, physical activity, as well as socio-economic impacts [27]. Other bacterial phyla such as the *Actinobacteria* phylum, which is comprised of the *Bifidobacterium* genus as well as other genera, can also play role in weight gain and obesity. Indeed, in an investigation of gut microbiota of lean and obese twins, higher levels of *Actinobacteria* were found in obese subjects [28].

The associations between body weight, weight loss and changes in major bacterial groups have not been studied up to now in populations of many countries around the world, including Ukraine. The aim of present study was to assess the differences in the composition of major phyla of gut microbiota in Ukraine adults with different BMI.

## Methods

### Study population

The fecal samples were obtained from 61 healthy adult individuals (mean age 44.2 years) during the period from March to May 2016 (Additional file 1: Table S1). These subjects were grouped into four groups on the basis of their BMI: those with a BMI <18.5 kg/m^2^ (underweight persons), those with a BMI between 18.5 and 24.9 kg/m^2^ (normal persons), those with a BMI between 25.0 and 29.9 kg/m^2^ (overweight persons), and those with a BMI ≥30.0 kg/m^2^ (obese persons). Exclusion criteria were: history of oncology or endocrinology disease, anorexia, psychiatric disorders, and acute relapse of any chronic disease. Demographic and lifestyle characteristics of studied subjects are presented in Table 1.

**Table 1:** Demographic and lifestyle characteristics of study population

Variable	BMI category			
	<18.5	18.5–24.9	25–29.9	≥30
	n (%)	n (%)	n (%)	n (%)
Age:				
20–39	7 (22.6)	18 (58.1)	3 (9.7)	3 (9.7)
40–59	-	6 (40.0)	7 (46.7)	2 (13.3)
> 60	-	3 (20.0)	6 (40.0)	6 (40.0)
Gender:				
Male	2 (13.3)	7 (46.7)	2 (13.3)	4 (26.7)
Female	5 (10.9)	20 (43.5)	14 (30.4)	7 (15.2)
Tobacco smoking:				
Never smoker	5 (21.7)	9 (39.1)	8 (34.8)	1 (4.3)
< 30 pack- years	2 (6.7)	15 (50.0)	6 (20.0)	7 (23.3)
≥ 30 pack- years	-	3 (37.5)	2 (25.0)	3 (37.5)
Physical activity:				
Sedentary (PAL 1.0–1.39)	1 (14.3)	2 (28.6)	2 (28.6)	2 (28.6)
Low active (PAL 1.4–1.59)	1 (9.1)	2 (18.2)	3 (27.3)	5 (45.5)
Active (PAL 1.6–1.89)	4 (12.5)	18 (56.3)	8 (25.0)	2 (6.3)
Very active (PAL 1.9–2.5)	1 (9.1)	5 (45.5)	3 (27.3)	2 (18.2)
Total	7 (11.5)	27 (44.3)	16 (26.2)	11 (18.0)

*PAL* physical activity level (a ratio of total energy expenditure to basal energy expenditure [33])

### Sample collection and DNA extraction

Fresh stool samples were provided by each subject in a stool container on site. Within 10 min upon defecation, the fecal sample was aliquoted and aliquots were immediately stored at 20 °C for 1 week until DNA isolation. DNA was extracted from 1.5–2 frozen stool aliquots using the phenol-chloroform method by protocol [29]. DNA was finally eluted in 200 μl elution buffer. The DNA quantity and quality was measured by NanoDrop ND-8000 (Thermo Scientific, USA). Samples with a DNA concentration less than 20 ng or an A 260/280 less than 1.8 were subjected to ethanol precipitation to concentrate or further purified, respectively, to meet the quality standards.

#### Oligonucleotide primers

Quantification of different taxa by qPCR using primers targeting the 16S rRNA gene, specific for *Firmicutes, Actinobacteria* and *Bacteroidet*es, as well as universal primers was performed. The primer sequences were:

*Bacteroidetes*:

798cfbF AAACTCAAAKGAATTGACGG (Forward).

and cfb967R GGTAAGGTTCCTCGCGCTAT (Reverse),

*Firmicutes*:

928F–firm TGAAACTYAAGGAATTGACG (Forward).

and 1040FirmR ACCATGCACCACCTGTC (Reverse),

*Actinobacteria*:

Act920F3 TACGGCCGCAAGGCTA (Forward).

and Act1200R TCRTCCCCACCTTCCTCCG (Reverse),

and universal bacterial 16S rRNA sequences:

926F AAACTCAAAKGAATTGACGG (Forward).

and 1062R CTCACRRCACGAGCTGAC (Reverse).

#### PCR amplification

PCR reaction was performed in real-time thermal cycler Rotor-Gene 6000 (QIAGEN, Germany). The PCR reaction conditions consisted of an initial denaturing step of 5 min at 95 °C, 30 cycles of 95 °C for 15 s, annealing for 15 s and 72 °C for 30 s, and a final elongation step at 72 °C for 5 min. Every PCR reaction contained 0.05 units/μl of Taq polymerase (Sigma Aldrich), 0.2 mM of each dNTP, 0.4 μM of each primer, 1× buffer, ~10 ng of DNA and water to 25 μl [30]. Samples were amplified with all primer pairs in triplicates. The Cts (univ and spec) were the threshold cycles registered by the thermocycler. The average Ct value obtained from each pair was transformed into percentage with the formula [28].

#### Identification of microbial composition

Determination of microbial composition at the level of major microbial phyla was carried out by identification of total bacterial DNA, and DNA of Bacteroidetes, Firmicutes and Actinobacteria was performed with quantitative real-time PCR (qRT-PCR), using gene-targeted primers.

### Statistical analysis

Statistical analysis was performed using the software STATISTICA 11.0. Shapiro–Wilk test was performed to test the normality of the distribution of all the quantitative variables studied. Since variables did not followed normal distribution, non-parametric methods were selected for further analysis of the data such as Spearman’s correlation and multivariate logistic regression. To identify the statistical difference among the BMI categories, median abundances of each phylum were compared by the Kruskal-Wallis test.

## Results

**Fig. 1:**
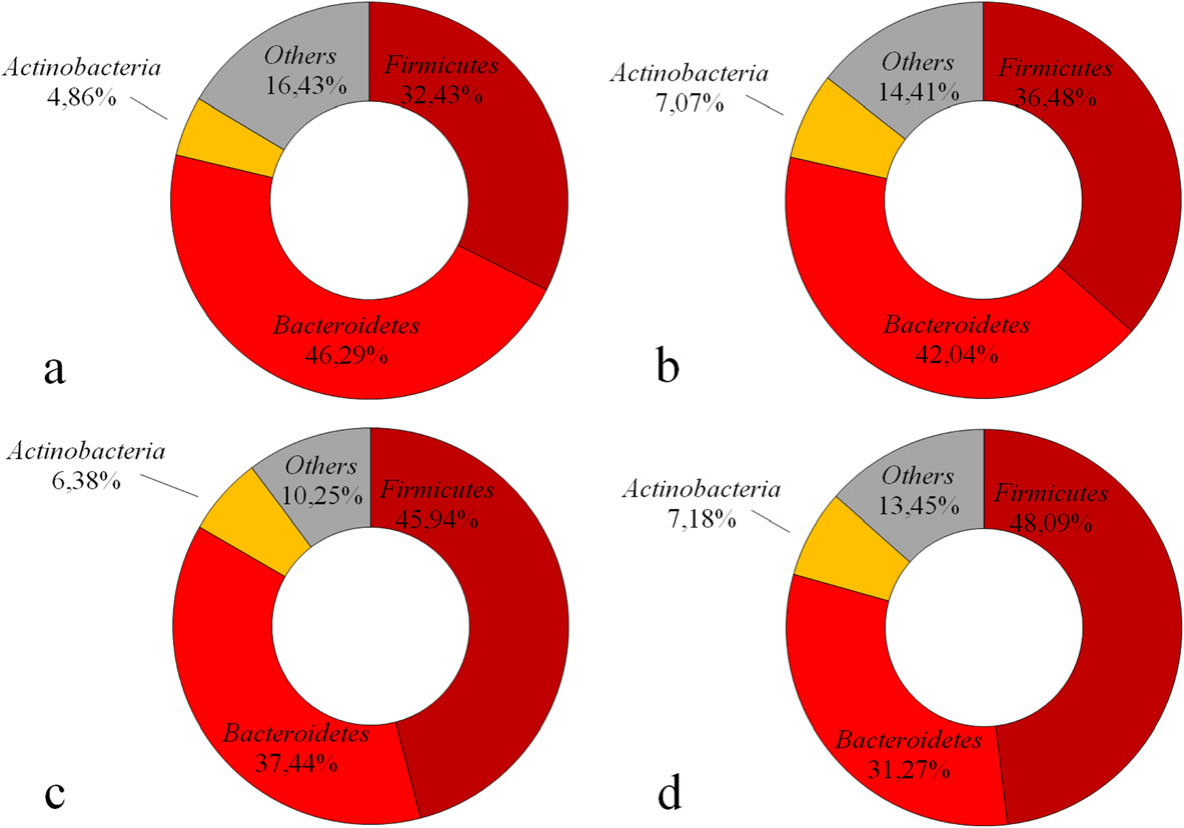
The relative abundance of the major microbial phyla in different BMI categories (**a** BMI < 18.5, **b** BMI 18.5–24.9, **c** BMI 25–29.9 and **d** BMI ≥30)

**Fig. 2:**
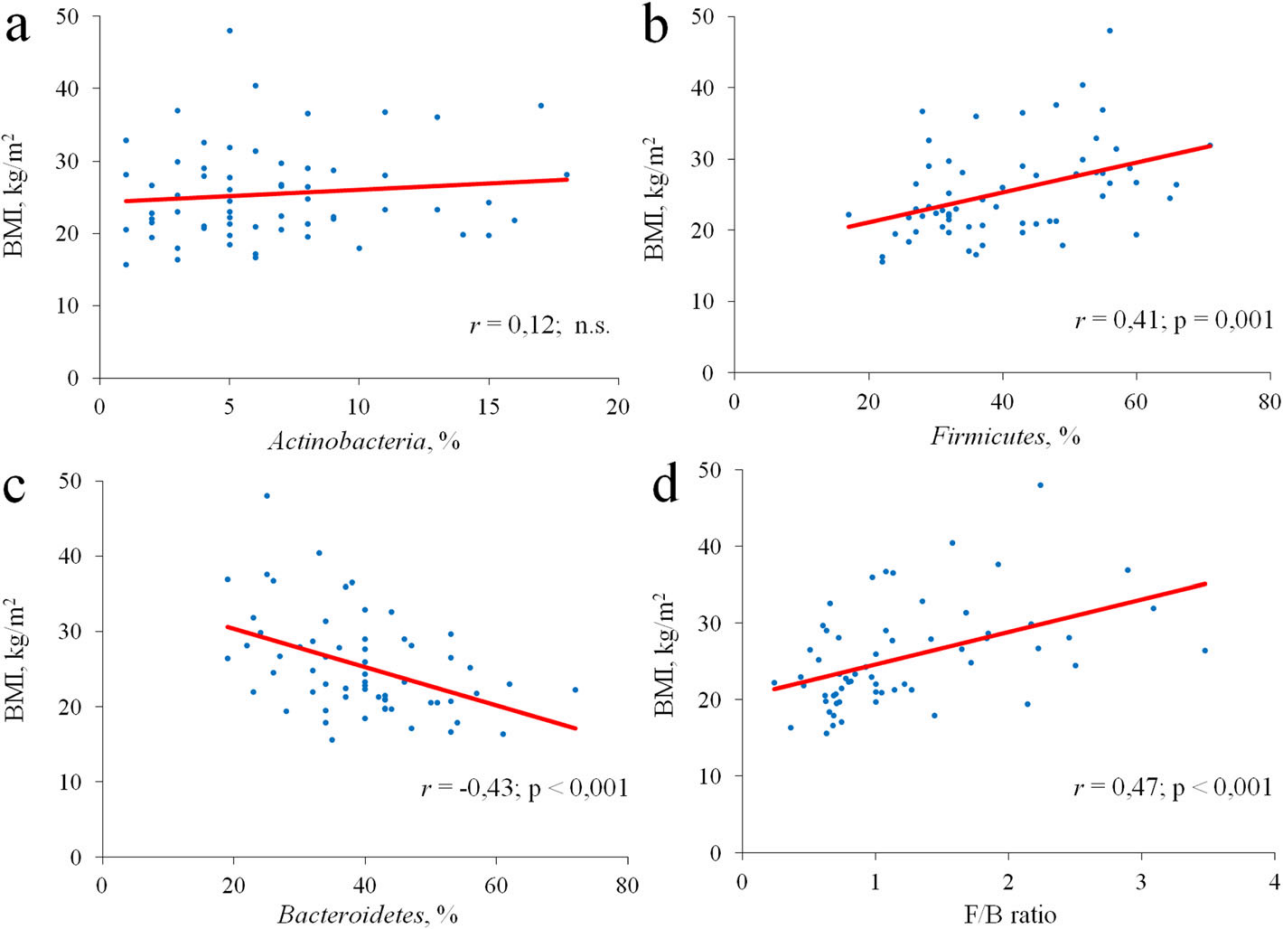
Regression plots of BMI against relative proportions of the main gut microbiota phyla. **a** *Actinobacteria,* **b** *Firmicutes*, **с** *Bacteroidetes* and **d** *Firmicutes/Bacteroidetes* ratio; n.s.: non-significant; *r*: Spearman’s correlation coefficient

The relative abundance of the major microbial phyla substantially varied between different BMI categories. The relative abundance of *Actinobacteria* was small (5–7%) and comparable in different BMI categories. The content of *Firmicutes* was gradually increased, while the content of *Bacteroidetes* was decreased with increasing BMI; the F/B ratio also raised with increasing BMI (Table 2, Figs. 1 and Fig. 2).

**Table 2:** Median abundance and interquartile range of each phylum across each of the BMI categories

Phylum	BMI category				*P*
	<18.5	18.5-24.9	25-29.9	≥30	
Actinobacteria	5 (3–6)	6 (4–9)	6 (3.5–8)	6 (4–11)	.707
Firmicutes	35 (22–37)	32 (29–43)	48 (33–56)	52 (36–56)	.010
Bacteroidetes	47 (35–54)	42 (34–46)	38 (29–47)	33 (25–38)	.016
F/B	0.7 (0.6–0.7)	0.8 (0.7–1.0)	1.3 (0.7–2.0)	1.6 (1.1–2.2)	.005

The lower and upper quartiles are given in parenthesis after the median values. To identify the statistical difference among the BMI categories, median abundances were compared by the Kruskal-Wallis test.

The age and sex composition was different in various BMI groups (see Table 1). So, they could be confounding factors and affect the association between F/B ratio and BMI. Therefore, the adjustment for these factors as well as for smoking and physical activity levels was performed by multivariate logistic regression model. In an unadjusted logistic regression model, F/B ratio was significantly associated with BMI. Those persons who had F/B ratio ≥ 1, were 23% more likely to be overweight than those who had F/B ratio < 1 (OR = 1.23, 95% CI 1,09–1,38, *p* < 0.0001). This association continued to be significant after adjusting for all confounders examined (OR = 1.33, 95% CI 1,11–1,60, *p* < 0.0001).

## Discussion

Consistently with many other studies [16–18, 31], we found significant increase in relative abundance of *Firmicutes* and higher F/B ratio in overweight and obese persons in Ukraine population. It is well known from many animal and human studies that obesity is associated with a composition of gut microbiota. There are some controversies, however, about significance of the F/B ratio, as well as about the impact of *Actinobacteria* level, on *the development of* obesity. A possible explanation for our findings is that *Firmicutes* are more effective as an energy source than *Bacteroidete*s, thus promoting more efficient absorption of calories and subsequent weight gain [5, 6]. In a study by Turnbaugh et al. [27] conducted in obese and lean twins, it has been shown that *Firmicutes* were dominant in the microbiomes of obese subjects, which were also enriched with genes known to be associated with nutrient transporters, while a higher relative abundance of *Bacteroidetes* and an enrichment of genes linked to carbohydrate metabolism was found in microbiomes of lean twins.

Results from several studies, however, are inconsistent with these findings. For example, the ratio of *Firmicutes* to *Bacteroidetes* was shifted in favor of the *Bacteroidetes* in overweight and obese subjects in the Schwiertz et al. [26] research. Another possible explanation for our findings could be the association between the relative proportion of gut anaerobic bacteria and blood glucose levels. Indeed, higher blood glucose levels were found to be negatively associated with relative proportion of *Bacteroides* in the gut of elderly people [32]. It seems important because higher blood glucose level is key component of metabolic syndrome. In addition, the characteristics of microbiome composition revealed in our study could, at least partly, be explained by the dietary habits in the Ukraine population. In particular, they likely can be attributed to the consumption of rye bread which is known to be more commonly eaten in Eastern Europe than in Western Europe, as well as of pork fat (“salo”). In future studies, we plan to investigate the dietary effects on the intestinal microbiota composition in the population of Ukraine.

One limitation of our study is that analyses were performed in stool samples, whereas the main part of nutrients is known to be absorbed in small intestine. The analysis of proximal gut microbiota may be more appropriate for investigation of the effects of gut bacteria on body weight and metabolic changes [21, 25]. These issues should be addressed in future research. In addition, we plan further investigation of the link between BMI and microbiome composition in Ukrainian population at the lower taxonomic levels.

## Conclusion

The data obtained in our study indicate that obese persons in Ukrainian adult population have a significantly higher level of *Firmicutes* and lower level of *Bacteroidetes* compared to normal-weight and lean adults. These findings from Ukraine population are consistent with findings from other populations.

## Additional file

Additional file 1: Table S1. Provides the individual data on the gender, age, anthropometric indices and relative abundance of the major microbial phyla in the study subjects. (XLSX 14 kb)

## Abbreviations

BMI: Body mass index
F/B ratio: *Firmicutes/Bacteroidetes* ratio
PAL: Physical activity level
qRT-PCR: Quantitative real-time PCR
